# Accuracy of wearable devices in predicting falls in older adults: a systematic review and meta-analysis

**DOI:** 10.3389/fpubh.2026.1778750

**Published:** 2026-03-11

**Authors:** Chuan Mou, Xiaoying Yan, Xinrui Miao, Liangyu Zhu

**Affiliations:** 1 Institute of Physical Education, Sichuan University, Chengdu, China; 2 School of Physical Education, Chengdu Normal University, Chengdu, China; 3 Institute of Physical Education, Kunsan National University, Gunsan, Republic of Korea

**Keywords:** fall prediction, meta-analysis, older adults, postural control, wearable devices

## Abstract

**Background:**

Wearable devices enable the continuous collection of kinematic information, such as gait and postural control, in real-life environments, offering potential for the early identification and stratified management of fall risk in older adults. However, quantitative integrated evidence regarding their overall accuracy in predicting future falls is lacking. This systematic review and meta-analysis aims to evaluate the accuracy of wearable devices in predicting falls among older adults and to explore the potential influence of key study characteristics on predictive performance.

**Methods:**

A systematic search was conducted in PubMed, Embase, Web of Science, and the Cochrane Library from database inception to October 9, 2025. Using a bivariate random-effects model, we pooled sensitivity and specificity, calculated likelihood ratios, and fitted a summary receiver operating characteristic (SROC) curve to determine the area under the curve (AUC). Subgroup analysis and meta-regression explored potential sources of heterogeneity. The risk of bias was assessed with the PROBAST tool.

**Results:**

A total of 20 studies were included. The pooled sensitivity was 0.55 (95% CI: 0.42–0.67), specificity was 0.89 (95% CI: 0.84–0.93), positive likelihood ratio was 5.2, negative likelihood ratio was 0.50, and diagnostic odds ratio was 10.39. The area under the summary receiver operating characteristic (SROC) curve was 0.85 (95% CI: 0.81–0.88). Subgroup and regression analyses indicated that studies employing machine learning modeling demonstrated superior overall discriminative ability (AUC = 0.90). Predictive performance may be influenced by factors such as population age structure, sample size, and sensor placement location.

**Conclusion:**

Wearable devices exhibit good discriminative ability for predicting future falls in older adults, characterized overall by high specificity and moderate sensitivity. They are more suitable as tools for early screening and risk stratification in community and institutional settings, thereby supporting decision-making regarding intervention priorities.

**Systematic review registration:**

https://www.crd.york.ac.uk/PROSPERO/view/CRD420251274570.

## 1 Introduction

Falls are a major cause of injury and a critical public health concern for older adults. The World Health Organization (WHO) defines a fall as “an event which results in a person coming to rest inadvertently on the ground or floor or other lower level.” As the second leading cause of unintentional injury deaths globally, falls account for roughly 684,000 fatalities each year, with the highest mortality occurring in those aged 60 years and older. Approximately 37.3 million severe falls necessitate medical attention annually (1). Given the ongoing trend of global population aging, this disease burden is rising and presents a central challenge for older adult health management and chronic care systems worldwide.

Falls result in short-term injuries such as abrasions and bruises, as well as more severe outcomes including fractures, dislocations, and concussions; these injuries increase hospitalization risk, accelerate functional decline, and raise the likelihood of disability. Falls also carry significant psychological and social consequences, most notably a “fear of falling.” This fear often leads to self-imposed activity restriction and reduced social engagement, initiating a detrimental cycle of “reduced activity-decreased physical capacity-increased risk of recurrent falls” (2–8). Such dynamics heighten the burden on patients and their families while placing sustained pressure on healthcare and social care systems. From a health economics perspective, falls impose a substantial economic burden, with annual medical expenditures costing the United Kingdom’s National Health Service approximately £2.3 billion (9) and the United States an estimated $50 billion (8). Consequently, given the steadily growing older adult population, the development of earlier and more precise strategies for fall risk identification and stratified management in community and long-term care settings represents a critical and urgent challenge for geriatric health promotion and preventive medicine.

Traditional fall risk assessment in older adults has primarily relied on clinical scales, fall history, and functional tests like the Timed Up and Go (TUG), Tinetti Assessment Tool, and Short Physical Performance Battery (SPPB) conducted in controlled settings (10–13). Although clinically feasible, these methods depend on subjective scoring or single-time-point measurements. They consequently often fail to capture the dynamic movement characteristics of daily life, and their accuracy in predicting future falls is inconsistent (14–17). Advances in micro-electromechanical systems (MEMS) and low-power sensing have progressively introduced wearable devices, especially inertial measurement unit (IMU)-based systems, into fall research. An IMU typically integrates accelerometers and gyroscopes to collect continuous, objective kinematic data in both laboratory and real-world environments. This capability supports the quantitative assessment of gait characteristics and postural control (10, 18, 19). Compared to traditional laboratory equipment, wearable devices are smaller, less expensive, highly scalable, and suitable for long-term monitoring. These advantages have established them as a mainstream technical solution in fall-related research over the past decade (20).

Early research using wearable devices primarily analyzed standardized gait tasks in controlled laboratory settings. By examining parameters such as walking speed, gait variability, and time- and frequency-domain acceleration features, these studies sought to distinguish fallers from non-fallers or to perform short-term risk assessments (18). Subsequent work deployed sensors in real-life scenarios to monitor gait performance continuously during older adults’ daily activities. This approach identified specific movement characteristics associated with the timing or frequency of subsequent falls (21–23). Data collected in natural environments may therefore offer greater ecological validity than laboratory tests, though their predictive capability remains inconsistent. As the dimensionality and complexity of wearable device data have increased, machine learning methods have been adopted for fall risk prediction. Studies have utilized models including Support Vector Machines (SVM), Random Forest (RF), Artificial Neural Networks (ANN), and deep learning to model multi-dimensional kinematic features, with some reporting high classification performance (24–28). These investigations, however, show considerable heterogeneity in sample size, follow-up design, feature extraction, and model validation strategies. The reported predictive performance consequently varies widely, and the generalizability of these models to broader populations of older adults requires further validation (29).

Research on wearable devices for fall detection has grown steadily in volume. Existing review articles and systematic reviews, however, focus predominantly on post-fall detection technologies, offering comparatively less comprehensive discussion of fall prediction or prevention studies (30–32). Several reviews explicitly note that most current work aims to achieve real-time identification and alerting for falls, a fundamentally passive response. In contrast, studies that prospectively predict future fall risk represent a notably smaller proportion of the literature, estimated at only about 12% (33–35). A systematic review and meta-analysis integrating existing research on wearable device-based fall prediction in older adults is therefore warranted to quantitatively evaluate overall predictive accuracy and to provide stronger evidence for applying this technology in fall risk screening and stratified management.

## 2 Methods

This systematic review and meta-analysis synthesizes studies on prediction accuracy. As all included studies reported binary outcomes (fall vs. non-fall) and permitted the construction of 2 × 2 contingency tables (true positive, false positive, true negative, false negative), we conducted our analysis and reporting in accordance with the Preferred Reporting Items for Systematic Reviews and Meta-Analyses for Diagnostic Test Accuracy Studies (PRISMA-DTA) (36).

### 2.1 Search strategy

A systematic search of the PubMed, Embase, Cochrane Library, and Web of Science databases was conducted. The search was restricted to English-language publications from each database’s inception until October 9, 2025. We employed a combination of Medical Subject Headings (MeSH) terms and free-text keywords, such as aged, elder, wearable sensor, fall, and predict. The complete search strategy is available in Supplementary Text 1. To ensure comprehensive literature retrieval, we also manually examined the reference lists of relevant systematic reviews and other identified articles.

### 2.2 Literature screening

#### 2.2.1 Inclusion criteria

- P (Population): The population comprised older adults (aged ≥ 60 years) from community, residential care, or healthcare settings, regardless of gender or comorbidities.
- E (Exposure): Eligible studies used wearable devices equipped with accelerometers, gyroscopes, or inertial measurement units to collect kinematic data for modeling future fall risk; sensors could be positioned on the trunk, waist, lower limbs, or multiple sites.
- C (Comparator): The comparator included different sensor configurations, prediction algorithms, or traditional fall risk assessment methods; studies without a formal comparator were also considered.
- O (Outcome): The required primary outcome was the predictive accuracy for falls during follow-up, with metrics such as sensitivity, specificity, or the raw counts necessary for their calculation (e.g., true positives) being directly extractable.
- S (Study design): We included prospective or retrospective observational prediction studies, while excluding those focused solely on fall detection or employing cross-sectional analyses.

#### 2.2.2 Exclusion criteria

- We excluded reviews, meta-analyses, conference abstracts, editorials, letters, case reports, commentaries, and brief surveys.
- Clinical trials, animal studies, and ex vivo studies were not eligible.
- Duplicate publications and studies for which the full text was inaccessible were also excluded.
- Finally, any literature from which outcome data could not be extracted was removed.

Two reviewers (XYY, XRM) independently screened the literature against the established criteria. Disagreements were resolved through discussion or by consulting a third reviewer (CM).

### 2.3 Data extraction and risk of bias assessment

Two reviewers (XYY and XRM) independently extracted data and assessed the risk of bias for each included study. The extracted information comprised the first author, publication year, country, study design, participants’ mean age, sex distribution, population source, the type and placement of wearable devices or sensors, the monitoring environment, follow-up duration, and the data analysis or prediction model employed. Outcome data were primarily extracted by constructing 2 × 2 contingency tables detailing true positives (TP), false positives (FP), false negatives (FN), and true negatives (TN). When these values were not directly reported, they were derived from the provided sensitivity, specificity, and sample size. If numerical data were still unavailable, sensitivity and specificity were estimated from published receiver operating characteristic (ROC) curves using digital extraction techniques, following established guidelines for diagnostic accuracy meta-analyses (37). The risk of bias and applicability of each included study were evaluated with the Prediction Model Risk of Bias Assessment Tool (PROBAST) (38). This tool assesses four domains, participants, predictors, outcome, and analysis through 20 signaling questions. A study was classified as having an overall high risk of bias if any single domain was rated high risk. Two reviewers performed all assessments independently, resolving disagreements through discussion or, when necessary, adjudication by a third reviewer (CM).

### 2.4 Data synthesis and statistical analysis

All statistical analyses were performed using Stata software (version 15.0; Stata Corporation, TX, United States). Pooled sensitivity, specificity, positive likelihood ratio (PLR), negative likelihood ratio (NLR), diagnostic odds ratio (DOR), and their corresponding 95% confidence intervals (CIs) were derived from a bivariate generalized linear mixed-effects model. We constructed a summary receiver operating characteristic (SROC) curve and estimated the area under the curve (AUC) to evaluate the overall discriminative ability of the predictive models. Between-study heterogeneity was assessed comprehensively through model parameters and the SROC curve’s shape, and was further quantified using Cochran’s Q test and the I^2^ statistic, with a *p* < 0.1 and an I^2^ > 50% indicating substantial heterogeneity. Subgroup analyses were conducted based on sample size, mean age, geographic region, population source, model type, and whether sensor placement included locations near the body’s center of mass. Potential sources of this heterogeneity were then explored via meta-regression analyses within the bivariate modeling framework. Publication bias was evaluated using Deeks’ funnel plot asymmetry test, where a *p* < 0.05 was considered indicative of significant bias.

## 3 Results

### 3.1 Study selection and flow diagram

**Figure 1:**
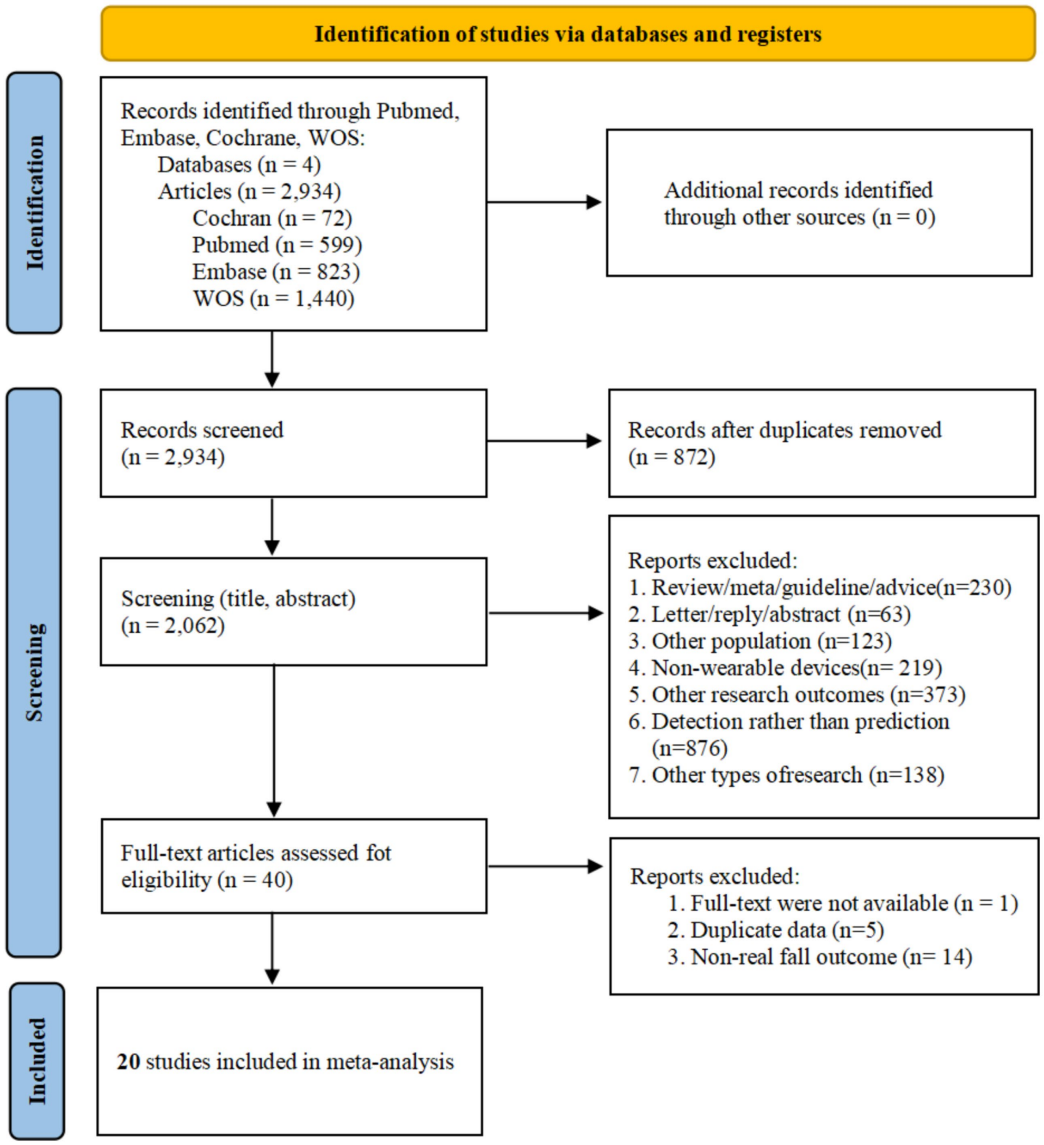
The PRISMA flowchart of the literature search and selection.

A total of 2,934 records were identified through database searches, with no additional studies found via reference list screening. Following duplicate removal, 2,062 records were screened by title and abstract. Of these, 2,022 records were excluded for failing to meet the inclusion criteria, leaving 40 articles for full-text eligibility assessment. Ultimately, 20 studies were included in the meta-analysis (39–58) (Figure 1).

### 3.2 Study characteristics and risk of bias assessment

**Figure 2:**
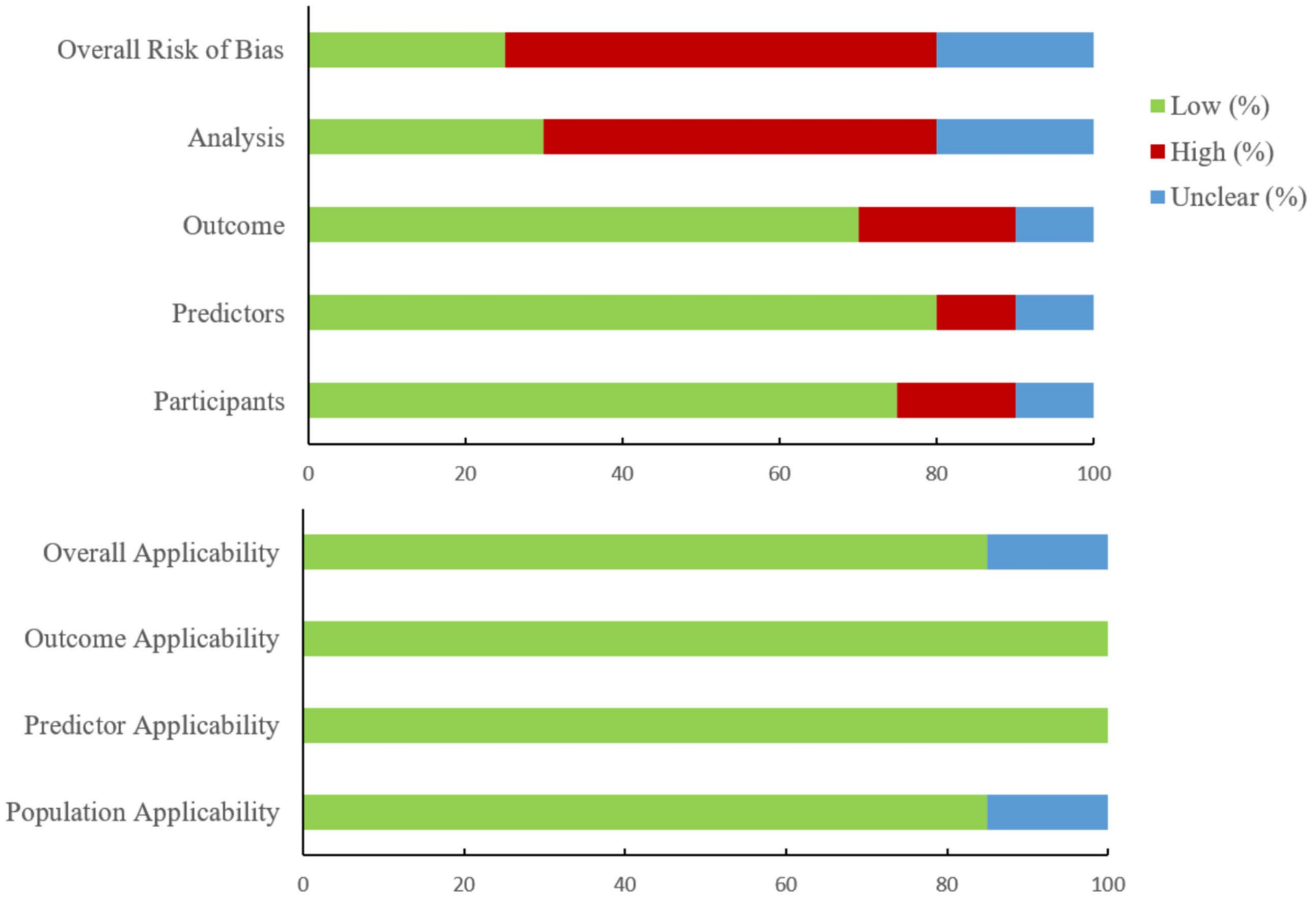
Risk of bias and applicability assessment of included studies using the PROBAST tool.

The 20 included studies (39–58) were conducted across 12 countries. Germany, the United States, and China contributed the largest number of studies (*n* = 3 each), while the remaining studies were from Japan (*n* = 1), the Netherlands (*n* = 2), the United Kingdom (*n* = 2), Canada (*n* = 1), the Czech Republic (*n* = 1), Pakistan (*n* = 1), Brazil (*n* = 1), Spain (*n* = 1), and Turkey (*n* = 1). All but one study, which employed a retrospective design (48), were prospective cohort studies. The study populations comprised 3,569 older adults, primarily recruited from community or healthcare settings, with mean ages ranging from 63.6 to 84.8 years. Fourteen studies reported sex distribution data that could be directly pooled, encompassing 1,343 male and 1,540 female participants; the remaining studies either did not report sex distribution or reported it in a manner not amenable to aggregation. All studies used wearable inertial sensing devices, primarily triaxial accelerometers or inertial measurement units, to collect kinematic data. Sensors were most commonly placed on the trunk (e.g., lower back or waist), though several studies employed multi-site configurations (e.g., lower limbs, pelvis, or head) to better capture gait and postural characteristics (Table 1). A risk of bias assessment using the PROBAST tool indicated a low risk of bias for most studies in the domains of participants, predictors, and outcomes, while the analysis domain was the primary source of uncertainty. The overall applicability of the included studies was deemed acceptable, with only a small number exhibiting unclear applicability concerns related to the participant domain (Figure 2).

**Table 1:** Characteristics of the included studies.

First author	Year	Country	Study design	*N* (Male/female)	Mean age (years)	Population source	Wearable device/sensor	Sensor placement	Monitoring setting	Follow-up (months)	Analysis model
Marschollek et al. (53)	2009	Germany	Prospective	110	Faller: 80.3 Non-faller: 80.0	Inpatients/outpatients	Tri-axial accelerometer	On the waist, near the center of mass	NR	NR	NR
Marschollek et al. (56)	2011	Germany	Prospective	46	81.3	Inpatients	Tri-axial accelerometer	On the waist	Real-world	12	Traditional statistical model
Doi et al. (57)	2013	Japan	Prospective	16/57	Faller: 84.8 ± 5.9 Non-faller:79.7 ± 8.2	Community-dwelling	Tri-axial accelerometer	Spinous processes at C7 (upper trunk) and L3 (lower trunk)	Real-world	12	Traditional statistical model
Kikkert et al. (45)	2017	The Netherlands	Prospective	20/41	Faller: 80.2 ± 4.7 Non-fallers:78.8 ± 5.1	Outpatients	Tri-axial accelerometer	Lower back	Real-world	8.6	Classical machine learning model
Howcroft et al. (55)	2018	Canada	Prospective	31/44	75.2 ± 6.6	Community-dwelling	Instrumented insole & Tri-axial accelerometer	Bilateral shanks, pelvis, and head	Real-world	6	Classical machine learning model
Bizovska et al. (49)	2018	Czech Republic	Prospective	23/108	70.8 ± 6.s7	Clubs	Tri-axial accelerometer	On the trunk near the L5 vertebra and on both shanks approximately 15 cm above the malleolus	Real-world	12	Traditional statistical model
Saadeh et al. (39)	2019	Pakistan	Prospective	12/8	65–70	NR	Tri-axial accelerometer	Upper thigh	Laboratory & Real-world	NR	Classical machine learning model
Lockhart et al. (54)	2021	USA	Prospective	Training Set: 127 Validation set: 44	Training Set: Faller: 75.44 ± 8.70 Non-faller: 75.77 ± 7.63 Validation set: Faller: 73.0 ± 8.1 Non-faller:75.8 ± 9.4	Community-dwelling	Inertial measurement unit	Sternum	Real-world	6	Classical machine learning model
Bet et al. (44)	2021	Brazil	Prospective	74	≥ 60	Community-dwelling	Tri-axial accelerometer	On the waist, near the center of mass	Real-world	12	NR
Ma et al. (46)	2022	China	Prospective	33/18	65.7 ± 8.4	Inpatients/ outpatients	Inertial measurement unit	NR	Real-world	6	Traditional statistical model
Kelly et al. (47)	2022	United Kingdom	Prospective	888/817	70	Community-dwelling	Tri-axial accelerometer	Hip-based	Real-world	12	Classical machine learning model
Dasgupta et al. (51)	2022	USA	Prospective	80/54	68.9 ± 8.1	Community-dwelling	Tri-axial accelerometer	Lower back	Real-world	3	NR
Ullrich et al. (42)	2023	Germany	Prospective	Faller: 6/4 Non-faller:20/5	Faller: 64.5 ± 8.2 Non-faller: 63.6 ± 8.4	Community-dwelling	Wearable inertial sensor	On each foot	Real-world	3	Classical machine learning model
Horak et al. (58)	2023	USA	Prospective	214	83 (80, 87)	Community-dwelling	Inertial measurement unit	On each foot, the low back, sternum and both wrists	Real-world	12	Bayesian model
Zhang Y. et al. (41)	2024	Netherlands	Prospective	84/179	71.8 ± 5.7	Community-dwelling	Tri-axial accelerometer	Lumbar L5	Real-world	6	Traditional statistical model
Zhang H. et al. (43)	2024	China	Prospective	Faller: 4/11 Non-faller:24/56	Faller: 85.1 ± 6.7 Non-faller:84.3 ± 8.3	Nursing home	Instrumented insole	feet	Real-world	NR	NR
Sotirakis et al. (52)	2024	United Kingdom	Prospective	Faller: 14/9 Non-faller:42/32	Faller: 71 (6.5) Non-faller: 65 (9.8)	Outpatients	Inertial measurement unit	Wrists, feet, sternum, and lumbar region	Real-world	5 yr	Classical machine learning model
Maiora et al. (50)	2024	Spain	Prospective	38/68	70–104	Nursing home	Inertial measurement unit	Below the waist,near the center of mass	Real-world	6	Deep learning model
Altunkaya et al. (48)	2024	Türkiye	Retrospective	71	78.36 ± 4.71	Community-dwelling	Tri-axial accelerometer	On the waist	Real-world	NR	Deep learning model
Lai et al. (40)	2025	China	Prospective	8/29	High-Risk Group:71.56 ± 5.01 Moderate-Risk Group:72.33 ± 3.80	Community-dwelling	Wearable inertial sensor	L5 vertebral level on the waist	Real-world	12	NR

Age is reported as presented in the original publications and may be shown as mean ± SD, median (IQR) or range. NR, not reported.

### 3.3 Meta-analysis results

**Figure 3:**
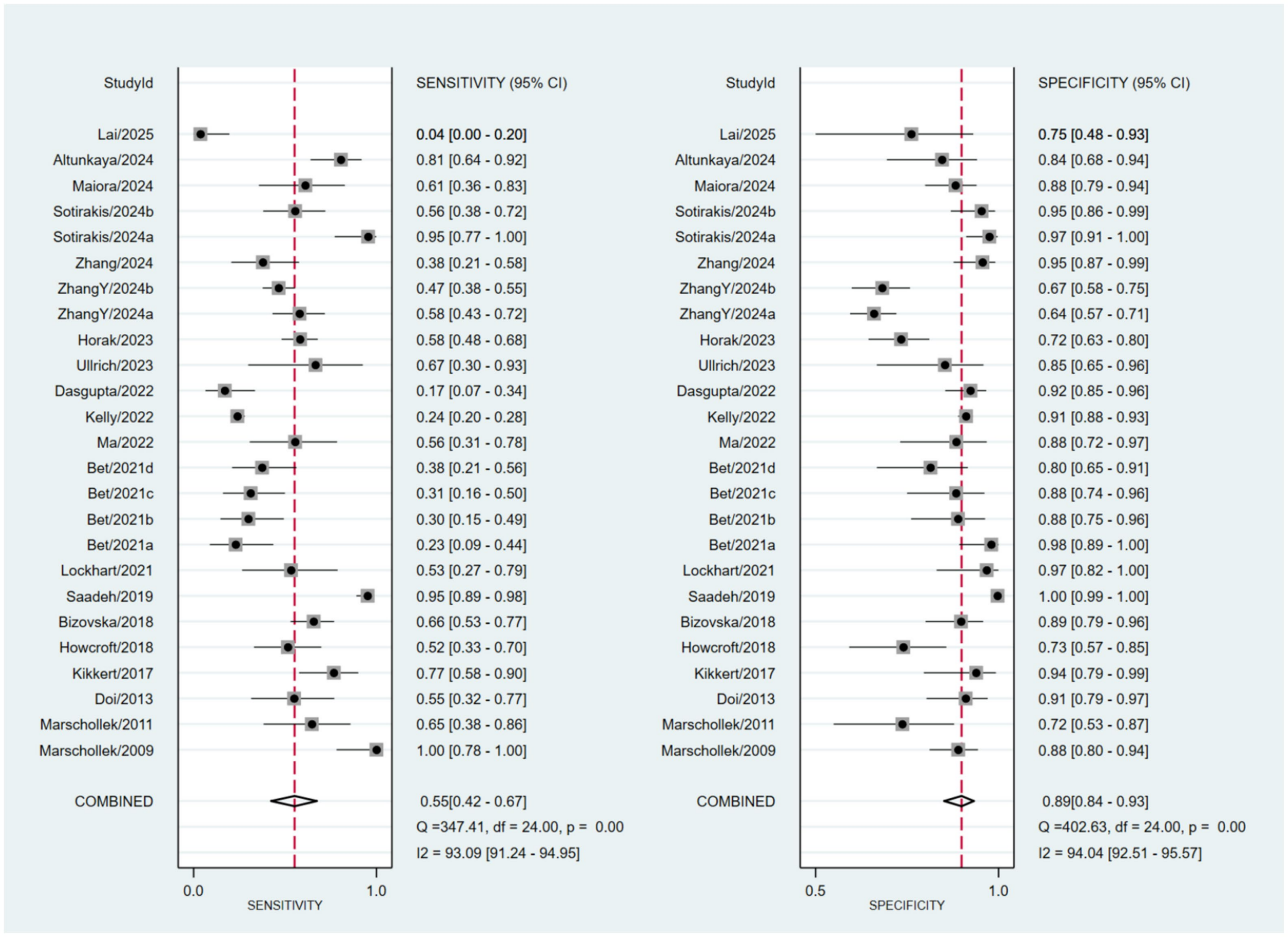
Forest plot of pooled sensitivity and specificity.

**Figure 4:**
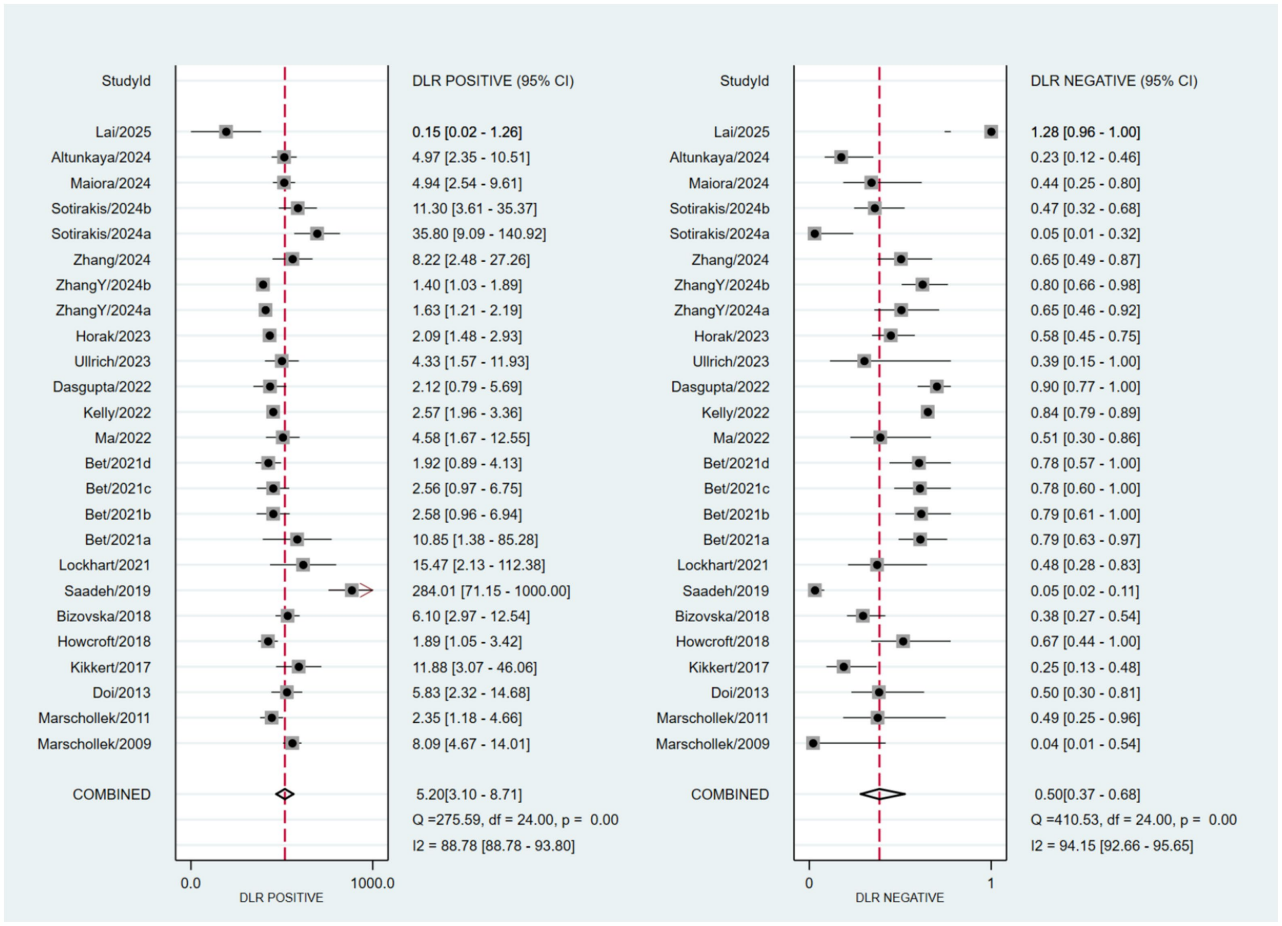
Forest plots of pooled positive and negative likelihood ratios.

**Figure 5:**
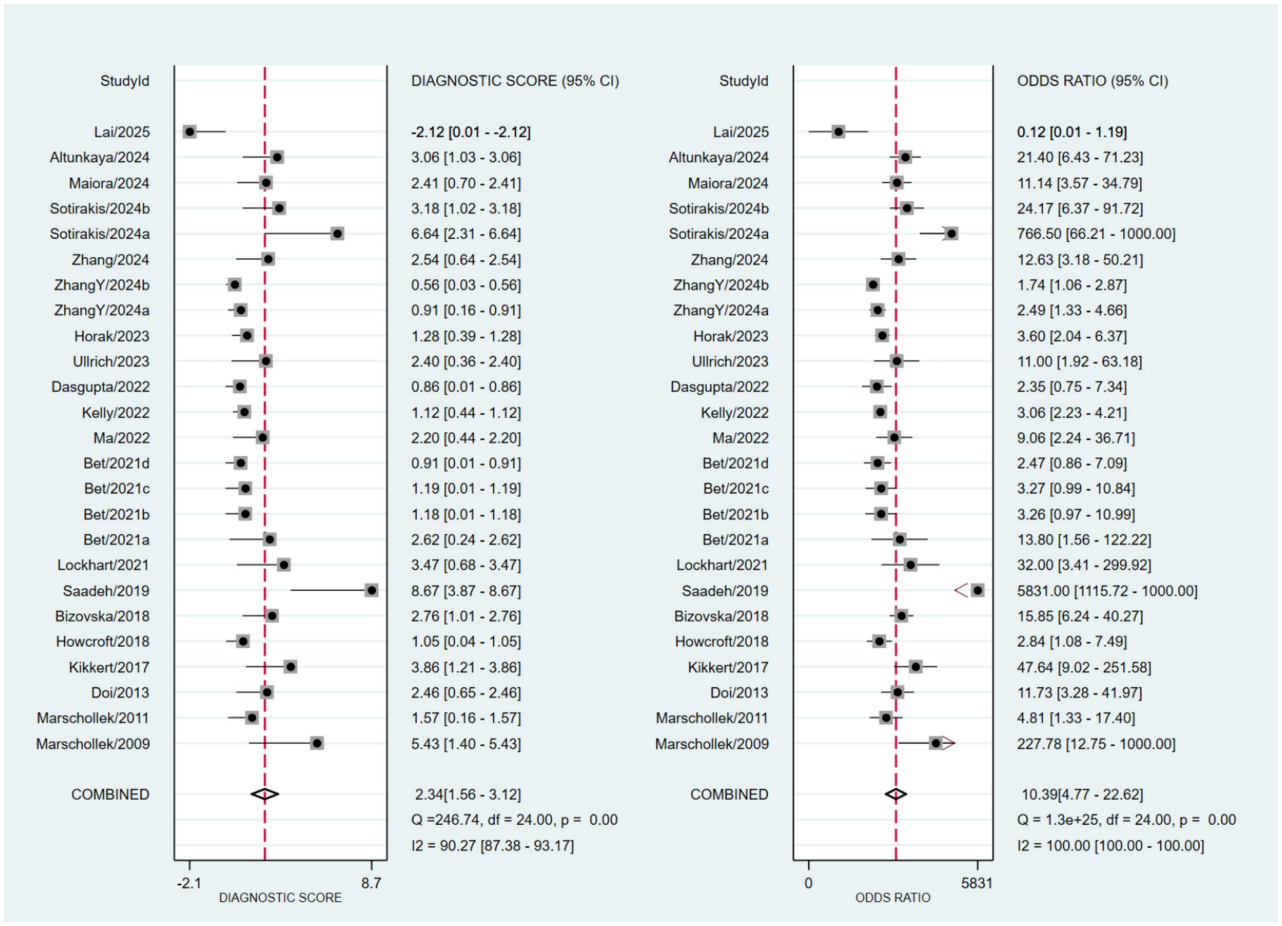
Forest plots of diagnostic score and diagnostic odds ratio.

**Figure 6:**
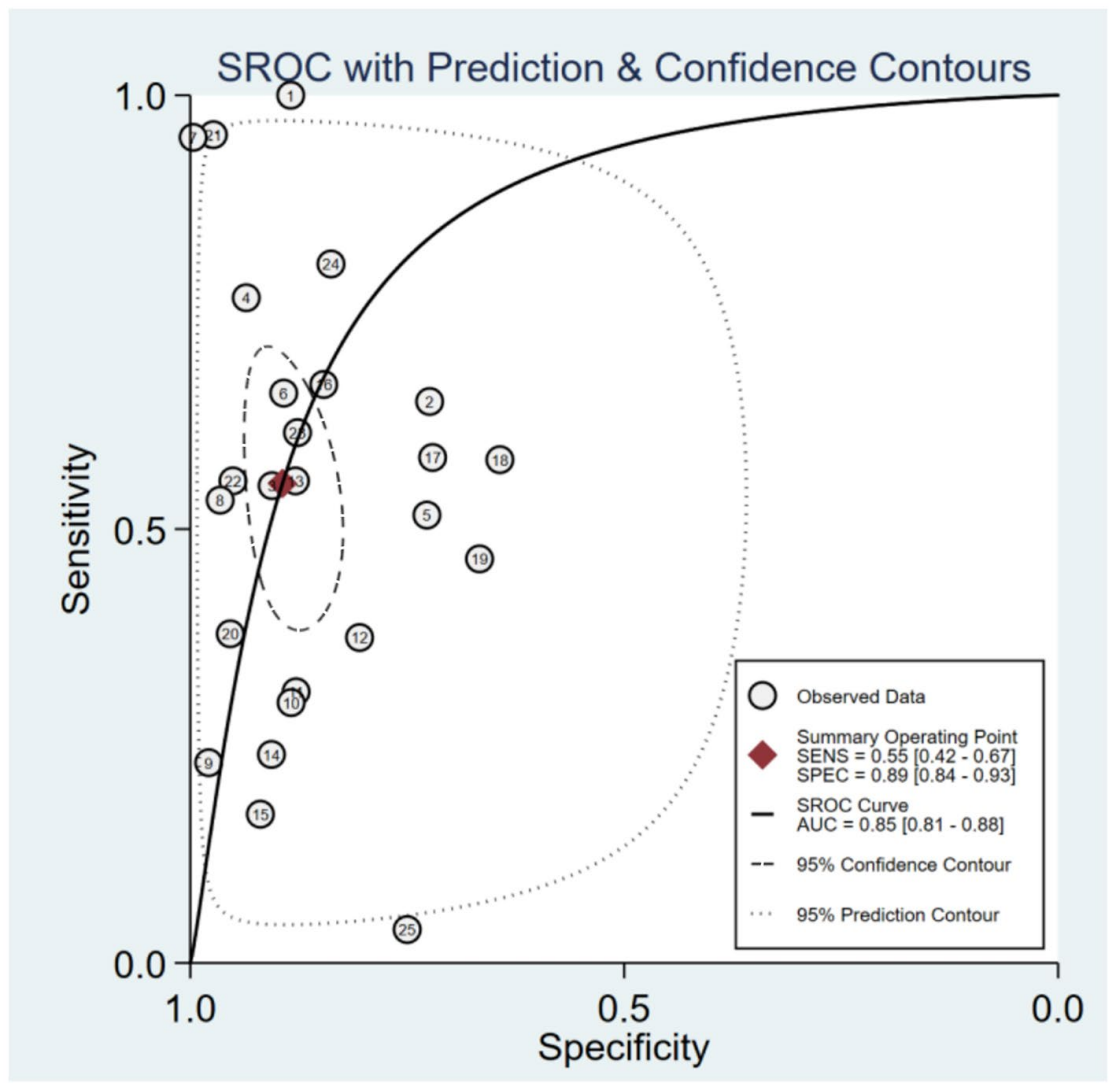
The SROC of sensitivity and specificity of wearable devices for fall risk prediction in older adults based on 20 studies.

Based on a bivariate generalized linear mixed-effects model, the pooled sensitivity of wearable devices for predicting falls in older adults was 0.55 (95% CI: 0.42–0.67; *I*^2^ = 93.09%), with a pooled specificity of 0.89 (95% CI: 0.84–0.93; *I*^2^ = 94.04%) (Figure 3). The corresponding pooled positive and negative likelihood ratios were 5.2 (95% CI: 3.1–8.71; *I*^2^ = 88.78%) and 0.5 (95% CI: 0.37–0.68; *I*^2^ = 94.15%), respectively (Figure 4). The pooled diagnostic odds ratio was 10.39 (95% CI: 4.77–22.62; *I*^2^ = 100.00%) (Figure 5). The SROC curve showed moderate dispersion of individual studies in the ROC space, with an overall area under the curve (AUC) of 0.85 (95% CI: 0.81–0.88) (Figure 6). This AUC value indicates that wearable devices have good discriminative performance for predicting fall risk in this population.

### 3.4 Subgroup and meta-regression analyses

Subgroup analyses were performed to investigate potential sources of heterogeneity according to prespecified covariates, with findings further assessed via meta-regression (Table 2). We evaluated the following factors: sample size (≥ 100 versus < 100), mean age (≥ 75 versus < 75 years), geographic region (Asia versus non-Asia), population source (community versus hospitalized/institutionalized), use of machine learning-based predictive models (yes versus no), and sensor configurations that included locations near the body’s center of mass, such as the trunk, waist, or hip (yes versus no).

**Table 2:** Results of univariable meta-regression and subgroup analysis.

Covariate	Category	Sensitivity (95% CI)	*P*1	Specificity (95% CI)	*P*2	PLR (95% CI)	NLR (95% CI)	DOR (95% CI)	AUC (95% CI)
Sample size	≥100	0.55 (0.33–0.76)	0.82	0.85 (0.77–0.94)	0.00*	3.5 (2.1–5.9)	0.55 (0.38–0.80)	6 (3–14)	0.80 (0.77–0.84)
	<100	0.56 (0.40–0.72)		0.91 (0.87–0.96)		6.5 (3.1–13.8)	0.49 (0.32–0.73)	13 (4–41)	0.87 (0.84–0.90)
Mean age	≥75	0.67 (0.48–0.85)	0.42	0.87 (0.79–0.95)	0.00*	4.9 (3.0–8.1)	0.40 (0.28–0.58)	12 (6–26)	0.85 (0.81–0.88)
	<75	0.48 (0.33–0.64)		0.90 (0.86–0.95)		5.1 (2.3–11.3)	0.57 (0.39–0.82)	9 (3–28)	0.83 (0.80–0.86)
Region	Asia	0.57 (0.32–0.83)	1	0.94 (0.88–0.99)	0.23	8.7 (1.7–43.9)	0.46 (0.19–1.13)	19 (2–218)	0.90 (0.87–0.92)
	Non-Asia	0.55 (0.40–0.69)		0.88 (0.83–0.93)		4.2 (2.8–6.5)	0.53 (0.41–0.69)	8 (4–15)	0.82 (0.79–0.86)
Population source	Community	0.52 (0.38–0.66)	0.33	0.90 (0.85–0.94)	0.14	5.1 (2.7–9.9)	0.53 (0.38–0.74)	10 (4–25)	0.83 (0.80–0.86)
	Inpatient/institutional	0.67 (0.41–0.93)		0.88 (0.78–0.98)		5.6 (3.5–9.1)	0.39 (0.21–0.72)	15 (5–38)	0.89 (0.86–0.91)
ML-based model	Yes	0.74 (0.61–0.87)	0.07	0.92 (0.88–0.97)	0.08	9.8 (3.8–25.2)	0.29 (0.18–0.47)	34 (9–137)	0.90 (0.87–0.92)
	No	0.42 (0.28–0.55)		0.87 (0.81–0.93)		3.1 (2.0–4.8)	0.68 (0.54–0.85)	5 (2–9)	0.79 (0.75–0.82)
Trunk-based sensors	Yes	0.51 (0.37–0.65)	0.23	0.88 (0.83–0.93)	0.00*	4.1 (2.6–6.6)	0.56 (0.41–0.76)	7 (3–16)	0.84 (0.80–0.87)
	No	0.68 (0.45–0.90)		0.93 (0.88–0.99)		9.8 (2.4–40.4)	0.34 (0.18–0.67)	28 (4–219)	0.88 (0.85–0.91)

CI, confidence interval; PLR, positive likelihood ratio; NLR, negative likelihood ratio; DOR, diagnostic odds ratio; AUC, area under the summary receiver operating characteristic curve; ML-based, machine learning–based prediction model. P1 and P2 are p values from meta-regression for subgroup differences in sensitivity and specificity, respectively, comparing the two levels of each covariate. An asterisk (*) indicates statistical significance at the prespecified level (e.g., *P* < 0.05). Data are pooled estimates from bivariate random-effects models for diagnostic test accuracy, stratified by study-level covariates (sample size, mean age, region, population source, model type, and trunk-based sensor placement).

#### 3.4.1 Sample size

Stratification by sample size revealed differences in predictive performance. Studies with smaller sample sizes (<100) demonstrated relatively higher specificity and overall discriminative ability, while those with larger sample sizes (≥100) exhibited more conservative performance (AUC: 0.87 vs. 0.80; specificity: 0.91 vs. 0.85). Meta-regression confirmed that sample size was significantly associated with specificity (regression *p* < 0.05), indicating it is a potential source of between-study heterogeneity.

#### 3.4.2 Mean age

In analyses stratified by mean age, studies with participants aged ≥75 years on average demonstrated superior overall predictive performance compared to those with a mean age below 75 years, primarily due to higher sensitivity and discriminative ability (sensitivity: 0.67 vs. 0.48; AUC: 0.85 vs. 0.83). Meta-regression confirmed a significant association between this age stratification and the observed differences in specificity (regression *p* < 0.05).

#### 3.4.3 Geographic region and population source

In subgroup analyses stratified by geographic region (Asia versus non-Asia) and population source (community-dwelling versus hospitalized or institutionalized), the overall differences in predictive performance were minor. Studies conducted in Asia showed a numerically higher discriminative ability (AUC: 0.90 vs. 0.82), while those involving hospitalized or institutionalized populations demonstrated slightly better predictive performance than community-based studies (AUC: 0.89 vs. 0.83). However, meta-regression analyses found no statistically significant association between these factors and either sensitivity or specificity.

#### 3.4.4 Machine learning-based predictive models

Machine learning algorithms significantly influenced predictive performance. Models incorporating machine learning demonstrated superior overall performance relative to those that did not, evidenced by higher sensitivity, diagnostic odds ratio, and discriminative ability (sensitivity: 0.74 vs. 0.42; DOR: 34 vs. 5; AUC: 0.90 vs. 0.79).

#### 3.4.5 Sensor placement

Subgroup analyses stratified by sensor placement revealed that trunk-based sensors achieved a pooled sensitivity of 0.51 (95% CI: 0.37–0.65) and a pooled specificity of 0.88 (95% CI: 0.83–0.93) for fall prediction. Non-trunk placements demonstrated higher performance, with a pooled sensitivity of 0.68 (95% CI: 0.45–0.90) and specificity of 0.93 (95% CI: 0.88–0.99). The difference in specificity between groups was statistically significant (*p* < 0.01), but the difference in sensitivity was not (*p* = 0.23). Studies using non-trunk sensors also yielded a higher diagnostic odds ratio (DOR: 28 vs. 7) and a larger area under the curve (AUC: 0.88 vs. 0.84).

Meta-regression analyses identified sample size, participant age structure, and the inclusion of sensors near the body’s center of mass as factors statistically associated with variations in predictive performance. The use of machine learning models consistently correlated with superior performance across multiple outcome measures. In contrast, geographic region and population source exerted relatively limited influence on predictive accuracy.

### 3.5 Clinical effect

**Figure 7:**
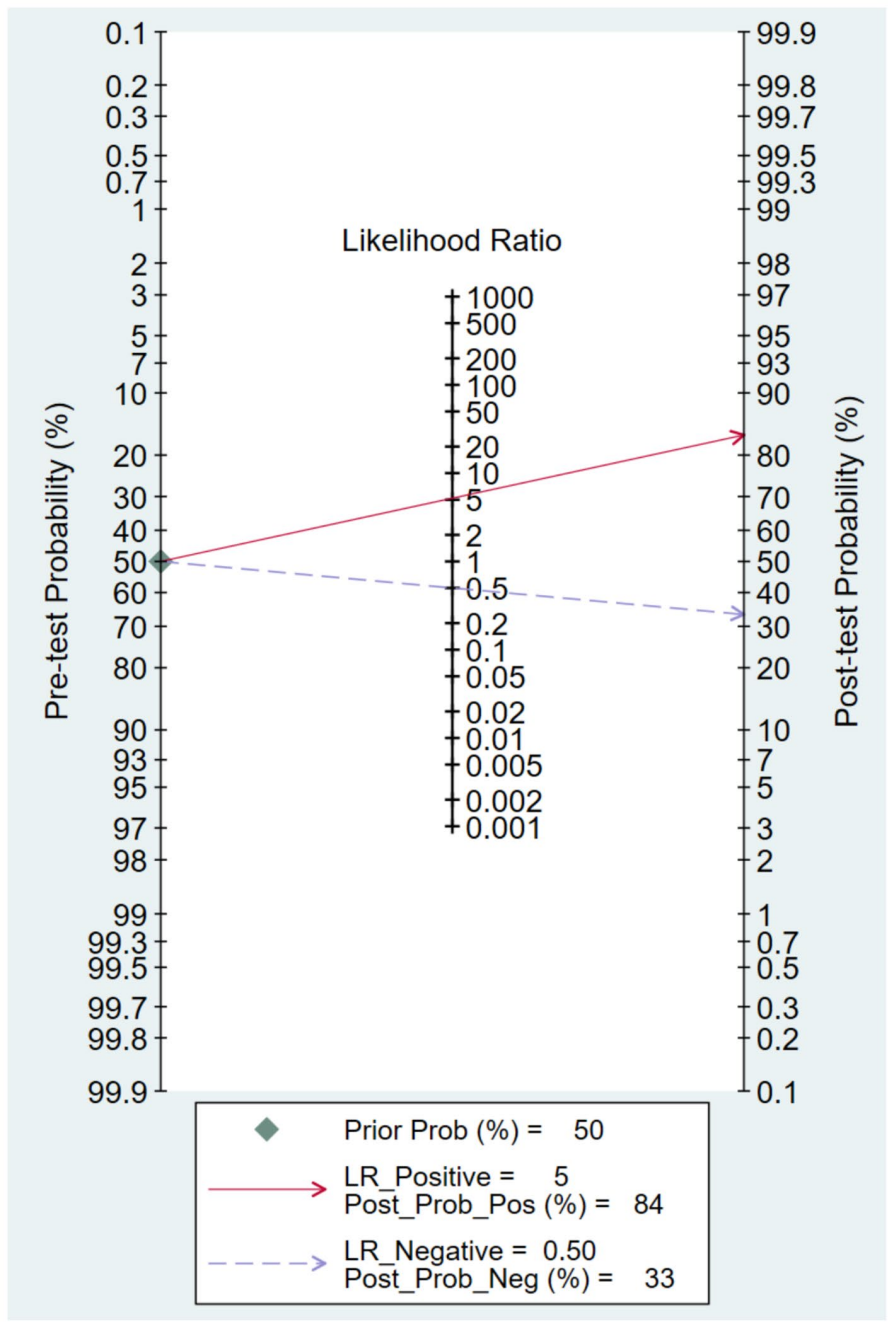
Fagan’s nomogram for clinical utility with 50% pretest probability.

To assess the clinical potential of wearable devices for predicting fall risk in older adults, a Fagan nomogram was developed using the pooled likelihood ratios (Figure 7). Given a pre-test probability of 50%, a positive prediction from the devices raised the post-test probability of a fall to 84%. In contrast, a negative prediction reduced the post-test probability to 33%.

### 3.6 Publication bias

**Figure 8:**
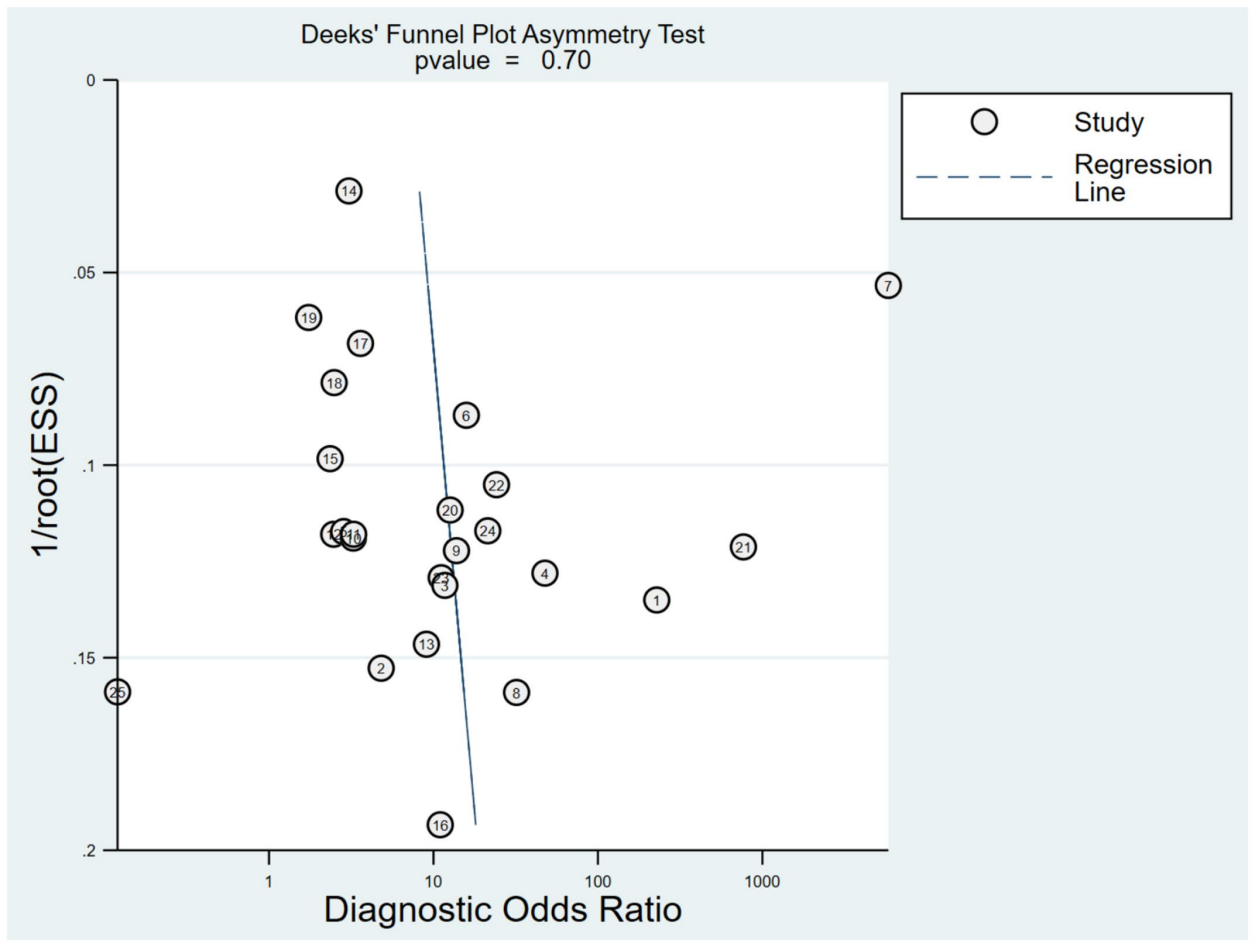
Deek’s funnel plot for publication bias analysis.

Publication bias was assessed using Deeks’ funnel plot asymmetry test (Figure 8). The results indicated no significant publication bias among the included studies (*p* = 0.70).

## 4 Discussion

This systematic review and meta-analysis synthesized evidence on predictive accuracy using a bivariate modeling framework. The pooled results indicate that wearable devices achieved a sensitivity of 0.55 (95% CI: 0.42–0.67) and a specificity of 0.89 (95% CI: 0.84–0.93) for predicting falls in older adults. The pooled AUC was 0.85 (95% CI: 0.81–0.88), reflecting good overall discriminative performance.

In this systematic review and meta-analysis, we found that despite substantial progress in fall-related technologies, most previous work has focused predominantly on fall detection rather than on fall prediction or prevention. Mubashir et al. classified detection approaches into wearable, ambient, and vision-based systems, but their review concentrated on technical implementation rather than on assessing future fall risk (30). Although the review by Delahoz and Labrador covered both detection and prevention, the authors explicitly noted persistent limitations in prediction, including heterogeneous evidence quality, insufficient real-world validation, and a lack of standardized methods (35). Likewise, a systematic review by Schwickert et al. on body-worn sensor-based fall detection confirmed that methodological inconsistencies, scarce real fall data, and non-uniform fall definitions continue to hinder study comparability and limit the generalizability of findings (59). Consequently, while these earlier reviews provide a necessary foundation for technological development, they do not adequately address a more clinically relevant question: how accurately wearable devices can predict future falls.

As research in the field increasingly focuses on risk assessment and prediction, Howcroft et al. suggested in their review that future studies should employ prospective designs to identify potential fallers, clarify the most clinically promising sensor placements, and link interpretable predictor variables to known fall risk factors (18). Sun and Sosnoff similarly observed in their systematic review that, despite the potential of various wearable technologies, high heterogeneity in measurement parameters, sensor placement, assessment tasks, and modeling methods prevents a robust consensus on their ability to predict future falls (60). Following a systematic review and meta-analysis, Montesinos et al. emphasized that discrepancies in sensor placement, assessment tasks, and extracted features are central to the difficulty of integrating existing evidence, which complicates practical application design (61). In an updated review, Bezold et al. further noted that most studies remain confined to laboratory walking tests, whereas continuous monitoring paradigms, such as long-term recording of sit-to-stand transitions and turns may yield more stable risk classification by better reflecting daily life (62). Chen et al., reviewing work focused on community-dwelling older adults, concluded that wearable-based fall risk assessment shows promise, though standardization and generalizability of findings remain significant obstacles (63). Ferreira et al. and Subramaniam et al. also summarized the field from perspectives including algorithm choice, validation procedures, assessment tasks, and sensor configuration, stressing that variations in validation strategies and real-world implementability substantially influence estimates of model performance (64, 65). In light of this emerging consensus, our systematic review and meta-analysis integrates multiple diagnostic performance metrics, including Se, Sp, PLR, NLR, DOR, and AUC, to provide quantitative evidence derived directly from pooled results. Despite considerable between-study heterogeneity, we obtained a pooled AUC estimate of 0.85. This result indicates that wearable-based fall prediction currently holds practical feasibility for use in risk-stratified management.

Based on the pooled results of this systematic review, subgroup analyses revealed key differences in model performance and their potential translational implications. Studies employing machine learning methods achieved significantly higher sensitivity, specificity, and AUC than those using non-machine learning approaches (AUC: 0.90 vs. 0.79), which aligns with earlier reviews (62, 64, 65). Wearable device signals are inherently high-dimensional, temporally correlated, and exhibit substantial inter-individual variability; machine learning models excel at extracting complex patterns like gait variability and inter-axis coupling, leading to superior discriminative performance (62, 64, 65). Within age subgroups, studies with a mean participant age ≥75 years demonstrated higher sensitivity and lower negative likelihood ratios, though specificity was slightly reduced. This likely reflects the more pronounced gait variability, diminished postural stability, and limited transitional movements in advanced age, which make fall-related anomalies easier to identify. However, multimorbidity and fluctuating functional status in this population can also increase false positives, thereby lowering specificity (66). Moreover, studies with sample sizes <100 yielded a higher pooled AUC than those with ≥100 participants, indicating a potential overfitting risk in smaller studies. This highlights the importance of future efforts to improve model robustness through expanded samples, multi-center collaboration, and external validation. Models developed for older adults in hospitals or long-term care settings generally outperformed those designed for community-dwelling populations. Institutionalized groups typically present more concentrated risk factors, greater functional limitation, and clearer signal separability. Community environments, by contrast, introduce high heterogeneity and data noise that can constrain model performance and generalizability. Regarding sensor placement, although the trunk or near the center of mass is often theorized to be more sensitive to postural stability changes (61), the present analysis found that studies using such placements did not achieve a higher pooled AUC (0.84 vs. 0.88). This outcome implies that the influence of sensor location may be moderated by other factors, including the assessment task type, the number of sensors, and the algorithmic approach.

In this study, most synthesized statistical indicators exhibited high heterogeneity. This heterogeneity arises from multiple sources, including potential differences in follow-up duration and fall outcome recording methods, inconsistencies in model construction strategies and risk determination thresholds, and insufficient transparency in study validation procedures and result reporting (59, 60, 62, 64). Consequently, while the observed heterogeneity does not inherently invalidate the conclusions, it underscores that the field remains in a phase of parallel exploration where a unified methodology has yet to be established. Future research should seek consensus on standardizing the definition of fall events and prospective recording criteria. It should also define a minimum set of reporting standards covering sensor placement, sampling frequency, assessment tasks, feature engineering, risk thresholds, and model validation and calibration procedures. Furthermore, actively promoting external validation studies using independent cohorts is essential. These steps would improve the comparability of evidence across studies and enhance the clinical generalizability and practical value of the findings (36, 67, 68).

This study has several limitations. First, the included studies exhibited substantial heterogeneity in participant characteristics, sensor types and placement, data collection protocols, model construction, and outcome assessment methods. Consistent with previous reviews in this field, the I^2^ values for several pooled estimates exceeded 90%, indicating considerable between-study variability, which may limit the stability and reliability of the pooled results. These findings should be interpreted with caution and in conjunction with the subgroup and meta-regression analyses. Second, several studies had relatively small sample sizes, were predominantly single-center, and lacked comprehensive external validation or model calibration, raising the possibility of model overfitting and performance overestimation. In addition, most studies focused on older adults without systematically accounting for common comorbidities, which may further limit the generalizability of the findings to more clinically complex populations. Finally, fall outcomes were primarily derived from long-term follow-up records, which are susceptible to underreporting and recall bias.

## 5 Conclusion

This systematic review and meta-analysis synthesized evidence from 20 studies, demonstrating that wearable devices exhibit strong discriminative ability for predicting falls in older adults, with a pooled AUC of 0.85. The performance was characterized by high specificity and moderate sensitivity, indicating these devices are suitable for early screening and risk stratification within this population. Consequently, they can help prioritize intervention resources. However, they are not recommended as a standalone method for ruling out future fall risk. Subgroup and regression analyses revealed that machine learning models performed better, and that predictive performance may be influenced by factors such as age structure, population source, and sensor configuration. Future research should focus on standardized, multi-center, large-sample prospective studies to strengthen external validation, improve calibration, and evaluate the clinical net benefit of real-world application.

## Data Availability

The original contributions presented in the study are included in the article/Supplementary material, further inquiries can be directed to the corresponding author.
