# Supplementary material for: Accuracy of wearable devices in predicting falls in older adults: a systematic review and meta-analysis
Source: Front Public Health. 2026 Mar 11;14:1778750. doi: 10.3389/fpubh.2026.1778750 (PMC13015825; doi:10.3389/fpubh.2026.1778750)
Supplement: Supplementary file 1 [file Table_1.DOCX]

# Supplementary Text 1 Literature search strategy

**1.Pubmed**

| Search number | Query |
| --- | --- |
| #1 | "Aged"[Mesh] |
| #2 | "Wearable Electronic Devices"[Mesh] |
| #3 | "Accidental Falls"[Mesh] |
| #4 | "aged"[Title/Abstract] OR "old"[Title/Abstract] OR "older"[Title/Abstract] OR "elder"[Title/Abstract] OR "elderly"[Title/Abstract] OR "senior citizen"[Title/Abstract] OR "senium"[Title/Abstract] OR "geriatric"[Title/Abstract] |
| #5 | "Wearable Electronic Devices"[Title/Abstract] OR "wearable*"[Title/Abstract] OR "wearable sensor"[Title/Abstract] OR "acceleromet*"[Title/Abstract] OR "IMU"[Title/Abstract] OR "gyroscop*"[Title/Abstract] OR "trunk accelerometry"[Title/Abstract] OR "smartwatch"[Title/Abstract] |
| #6 | "Falling"[Title/Abstract] OR "fall"[Title/Abstract] OR "Falls"[Title/Abstract] |
| #7 | "predict*"[Title/Abstract] OR "Forecast*"[Title/Abstract] OR "prediction model"[Title/Abstract] OR "AUC"[Title/Abstract] OR "sensitivity"[Title/Abstract] OR "specificity"[Title/Abstract] OR "machine learning"[Title/Abstract] OR "deep learning"[Title/Abstract] OR "algorithm"[Title/Abstract] OR "model"[Title/Abstract] OR "random forest"[Title/Abstract] |
| #8 | ("Aged"[Mesh]) OR ("aged"[Title/Abstract] OR "old"[Title/Abstract] OR "older"[Title/Abstract] OR "elder"[Title/Abstract] OR "elderly"[Title/Abstract] OR "senior citizen"[Title/Abstract] OR "senium"[Title/Abstract] OR "geriatric"[Title/Abstract]) |
| #9 | ("Wearable Electronic Devices"[Mesh]) OR ("Wearable Electronic Devices"[Title/Abstract] OR "wearable*"[Title/Abstract] OR "wearable sensor"[Title/Abstract] OR "acceleromet*"[Title/Abstract] OR "IMU"[Title/Abstract] OR "gyroscop*"[Title/Abstract] OR "trunk accelerometry"[Title/Abstract] OR "smartwatch"[Title/Abstract]) |
| #10 | ("Accidental Falls"[Mesh]) OR ("Falling"[Title/Abstract] OR "fall"[Title/Abstract] OR "Falls"[Title/Abstract]) |
| #11 | ((("predict*"[Title/Abstract] OR "Forecast*"[Title/Abstract] OR "prediction model"[Title/Abstract] OR "AUC"[Title/Abstract] OR "sensitivity"[Title/Abstract] OR "specificity"[Title/Abstract] OR "machine learning"[Title/Abstract] OR "deep learning"[Title/Abstract] OR "algorithm"[Title/Abstract] OR "model"[Title/Abstract] OR "random forest"[Title/Abstract]) AND (("Aged"[Mesh]) OR ("aged"[Title/Abstract] OR "old"[Title/Abstract] OR "older"[Title/Abstract] OR "elder"[Title/Abstract] OR "elderly"[Title/Abstract] OR "senior citizen"[Title/Abstract] OR "senium"[Title/Abstract] OR "geriatric"[Title/Abstract]))) AND (("Wearable Electronic Devices"[Mesh]) OR ("Wearable Electronic Devices"[Title/Abstract] OR "wearable*"[Title/Abstract] OR "wearable sensor"[Title/Abstract] OR "acceleromet*"[Title/Abstract] OR "IMU"[Title/Abstract] OR "gyroscop*"[Title/Abstract] OR "trunk accelerometry"[Title/Abstract] OR "smartwatch"[Title/Abstract]))) AND (("Accidental Falls"[Mesh]) OR ("Falling"[Title/Abstract] OR "fall"[Title/Abstract] OR "Falls"[Title/Abstract])) |

**2.Cochrane**

| Search number | Query |
| --- | --- |
| #1 | MeSH descriptor: [Aged] explode all trees |
| #2 | MeSH descriptor: [Wearable Electronic Devices] explode all trees |
| #3 | MeSH descriptor: [Accidental Falls] explode all trees |
| #4 | (aged):ti,ab,kw OR (old):ti,ab,kw OR (older):ti,ab,kw OR (elder):ti,ab,kw OR (elderly):ti,ab,kw OR (senior citizen):ti,ab,kw OR (senium):ti,ab,kw OR (geriatric):ti,ab,kw |
| #5 | (Wearable Electronic Devices):ti,ab,kw OR (wearable*):ti,ab,kw OR (wearable sensor):ti,ab,kw OR (acceleromet*):ti,ab,kw OR (IMU):ti,ab,kw OR (gyroscop*):ti,ab,kw OR (trunk accelerometry):ti,ab,kw OR (smartwatch):ti,ab,kw |
| #6 | (Falling):ti,ab,kw OR (fall):ti,ab,kw OR (Falls):ti,ab,kw |
| #7 | (predict*):ti,ab,kw OR (Forecast*):ti,ab,kw OR (prediction model):ti,ab,kw OR (AUC):ti,ab,kw OR (sensitivity):ti,ab,kw OR (specificity):ti,ab,kw OR (machine learning):ti,ab,kw OR (deep learning):ti,ab,kw OR (algorithm):ti,ab,kw OR (model):ti,ab,kw OR (random forest):ti,ab,kw |
| #8 | #1 or #4 |
| #9 | #2 or #5 |
| #10 | #3 or #6 |
| #11 | #7 and #8 and #9 and #10 |

**3. Web of science**

| Search number | Query |
| --- | --- |
| #1 | TS=(aged) OR TS=(old) OR TS=(older) OR TS=(elder) OR TS=(elderly) OR TS=(senior citizen) OR TS=(senium) OR TS=(geriatric) |
| #2 | TS=(Wearable Electronic Devices) OR TS=(wearable*) OR TS=(wearable sensor) OR TS=(acceleromet*) OR TS=(IMU) OR TS=(gyroscop*) OR TS=(trunk accelerometry) OR TS=(smartwatch) |
| #3 | TS=(Falling) OR TS=(fall) OR TS=(Falls) |
| #4 | TS=(predict*) OR TS=(Forecast*) OR TS=(prediction model) OR TS=(AUC) OR TS=(sensitivity) OR TS=(specificity) OR TS=(machine learning) OR TS=(deep learning) OR TS=(algorithm) OR TS=(model) OR TS=(random forest) |
| #5 | #4 AND #3 AND #2 AND #1 |

**4. Embase**

| Search number | Query |
| --- | --- |
| #1 | 'aged'/exp |
| #2 | 'wearable device'/exp |
| #3 | 'falling'/exp |
| #4 | 'aged':ab,ti OR 'old':ab,ti OR 'older':ab,ti OR 'elder':ab,ti OR 'elderly':ab,ti OR 'senior citizen':ab,ti OR 'senium':ab,ti OR 'geriatric':ab,ti |
| #5 | 'wearable electronic devices':ab,ti OR 'wearable*':ab,ti OR 'wearable sensor':ab,ti OR 'acceleromet*':ab,ti OR 'imu':ab,ti OR 'gyroscop*':ab,ti OR 'trunk accelerometry':ab,ti OR 'smartwatch':ab,ti |
| #6 | 'falling':ab,ti OR 'fall':ab,ti OR 'falls':ab,ti |
| #7 | 'predict*':ab,ti OR 'forecast*':ab,ti OR 'prediction model':ab,ti OR 'auc':ab,ti OR 'sensitivity':ab,ti OR 'specificity':ab,ti OR 'machine learning':ab,ti OR 'deep learning':ab,ti OR 'algorithm':ab,ti OR 'model':ab,ti OR 'random forest':ab,ti |
| #8 | #1 OR #4 |
| #9 | #2 OR #5 |
| #10 | #3 OR #6 |
| #11 | #7 AND #8 AND #9 AND #10 |
